# Charting the Interplay between Structure and Dynamics in Complex Networks

**DOI:** 10.1371/journal.pbio.0030369

**Published:** 2005-10-04

While intelligent-design proponents enjoy their 15 minutes of fame denying the role of evolutionary forces in generating complex networks in nature, scientists are probing the organizing principles that govern these networks. Traditional models of complex networks assumed that connections between units—such as genes, proteins, neurons, or species—occur randomly. These notions changed as studies of protein interaction networks and other biological systems revealed “small world” features—characterized by short paths between nodes and highly clustered connections—and varying levels of organization, with certain patterns of local connections occurring more frequently in complex networks than in random networks. What determines the abundance of these so-called network motifs in specific networks is not known.

To study complex, dynamic systems, researchers create graphical representations with network maps. Though the network structure is static, the nodes represent values that change with time. For example, the neural map of the worm Caenorhabditis elegans contains 302 neurons (represented as nodes) and roughly 7,000 synaptic connections that appear fixed even though they represent transient behavior, such as activation states of individual neurons and their probable interactions. Discerning the global dynamics of these network structures has proved a major challenge.

In a new study, Robert Prill, Pablo Iglesias, and Andre Levchenko use the power of computational analysis to tackle the problem of identifying the dynamic features of a large-scale network. They found a high correlation between a dynamic property of a network motif—ability to withstand small fluctuations in the system—and its relative abundance in well-characterized biological networks. Their results suggest that just as connections between individual components of a biological network—be they genes, proteins, or cells—influence function, the dynamic properties of a network motif relate to the motif's function and could determine its prevalence in biological networks. [Fig pbio-0030369-g001]

**Figure pbio-0030369-g001:**
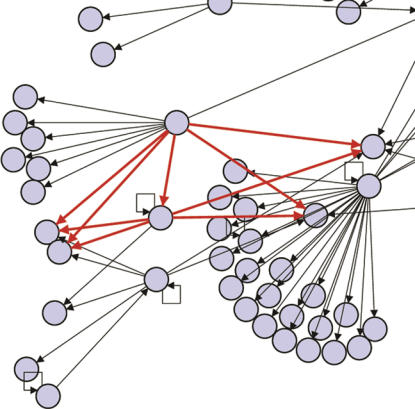
In this schematic of a transcriptional network, nodes represent operons (a set of bacterial structural genes and their regulatory elements) and links represent transcription factor DNA binding interactions. (Image generated with Cytoscape v1.1)

For a network motif to qualify as stable, it must return to steady state after small-scale perturbations, defined as intrinsic random fluctuations, or noise, and transient oscillations in activity. The behavior of a motif is determined by the direction, sign (presence of positive or negative feedback loops), and strength of the connections. The authors varied these parameters to simulate motif response to small perturbations. To measure stability, the authors assigned a structural stability score (SSS) as the probability that a particular motif returns to a postperturbation steady state. They use this metric to analyze the dynamics of all possible three- or four-node networks (noting that even two-node networks exhibit complex behavior). Based on the SSS scores, all the structurally distinct three- and four-node network motifs fell into three distinct categories: robustly stable circuits with no feedback loops, moderately stable circuits with a single two-node feedback loop (assuming a negative feedback loop), and, least stable, a mixture of complicated, highly connected motifs.

Comparing motif abundance in known biological networks with the SSS scores of simulated motifs revealed an “excellent correlation” between stability and motif abundance. Higher stability motifs were more abundant in the real networks, while low-stability motifs were absent, suggesting that the nonrandom character of network organization is driven by the structural stability of network motifs. To see how these motifs might operate as functional units, the authors used microarray data from yeast subjected to five different environmental stresses, including mild heat shock and hydrogen peroxide treatment, and mapped activated genes (represented as nodes) to their locations in the network. All the active regulatory motifs had a high stability score, suggesting that the nonrandom nature of the yeast transcriptional network may have arisen from selection acting on small motifs that respond robustly to specific environmental stresses. Expanding their analysis to other biological networks, the authors found that yeast and the pathogen Escherichia coli have similar motif profiles, likely reflecting similar environmental pressures, while the fruit fly transcription program and worm neuron network contain different motifs, reflecting both different environmental and functional demands.

These results suggest that both global constraints on the network and properties of network motifs themselves influence the abundance of motifs and the overall structure of a given network. While the authors caution that their networks are stripped-down versions of those found in biological systems, they point out that their approach can incorporate more complicated interactions as understanding of living networks increases. And with this new understanding, scientists can test the hypothesis that selective pressures favor motifs with particular dynamic properties. For more information on structural stability and networks, please see the accompanying Primer by Doyle (DOI: 10.1371/journal.pbio.0030392). —*Liza Gross*

